# An Autologous, Vascularized and Immunocompetent Tissue Engineered Skin to Highlight Inter‐Individual Variability to Better Understand the Human Wound Healing

**DOI:** 10.1111/wrr.70129

**Published:** 2026-01-29

**Authors:** Emilie Attiogbe, Elodie Mareux, Sébastien Larochelle, Adèle Mauroux, Sandrine Gofflo, Carine Mainzer, Sylvie Bordes, Brigitte Closs, Caroline Gilbert, Véronique J. Moulin

**Affiliations:** ^1^ Department of Surgery, Faculty of Medicine Université Laval Québec Quebec Canada; ^2^ Axe Médecine Régénératrice Centre de Recherche du CHU de Québec‐Université Laval Québec Quebec Canada; ^3^ R&D Department SILAB Brive France; ^4^ Département de Microbiologie‐Infectiologie et D'immunologie, Faculté de Médecine Université Laval Québec Quebec Canada; ^5^ Axe Maladies Infectieuses et Immunitaires Centre de Recherche du CHU de Québec‐Université Laval Québec Quebec Canada

**Keywords:** 3D model, autologous, immune cells, inter‐donor variability, microvasculature, skin, tissue engineering, wound healing

## Abstract

In vitro tissue‐engineered skin models are valuable tools for dermatological research. Yet, they often fail to reproduce the complex interactions among vascular, immune, and cutaneous cells during the wound healing process. In addition, inter‐individual variability response remains poorly understood, limiting our knowledge of the genetic and environmental factors that influence wound healing. This study presents a 3D autologous, vascularised, and immunocompetent Tissue Engineered Skin model (aviTES), generated using cells from the same donor to study the wound healing process. The aviTES models were injured using a 2 mm punch biopsy, and wound closure was macroscopically monitored for 7 days in the absence of any stimuli. The re‐epithelialisation rate was highly reproducible within the same donor. However, wound closure differed between healthy donors (five distinct donors), highlighting individual variability. Indeed, in most cases, complete re‐epithelialisation was observed within 2–4 days, but one did not close completely after 7 days. Immunofluorescence analysis revealed lymphocytes (CD45+/CD3+) migration for all donors, but no migration of CD206‐positive cells or neo‐angiogenesis into the wound sites. By contrast, platelet lysate treatment promoted epidermal cell migration, capillary organisation/neo‐angiogenesis, and altered collagen and MMP secretion at levels that were specific to each donor, highlighting donor‐specific responses to treatment. This innovative autologous skin model reproduced several key features of the human wound healing process and may represent a new tool for studying wound healing process and inter‐individual variability, paving the way for advances in personalised medicine.

**Abbreviations**
aviTES: autologous, vascularised and immunocompetent tissue engineered skin
BEC: blood microvascular endothelial cells
LEC: lymphatic microvascular endothelial cells
MMP: matrix metalloproteinases
PL: platelet lysate
PRP: platelet‐rich plasma

## 1 Introduction

Wound healing involves coordinated interactions between the cutaneous, vascular and immune systems [1, 2, 3]. Keratinocytes and fibroblasts contribute to skin structure and repair. Lymphatic and blood microvascular endothelial cells and resident or circulating immune cells regulate inflammation, angiogenesis and matrix remodelling [4]. Macrophages and myofibroblasts engage in feedback loops that influence healing [5, 6]. Similarly, fibroblasts stabilise vessels and produce structural matrix [7]. Endothelial cells recruit immune cells to the site of injury, while fibroblasts modulate the inflammatory environment by releasing cytokines and other signalling molecules [8]. Conversely, chronic wounds arise from the persistence of the inflammatory phase, which hinders the initiation of the proliferative and remodelling phases. They are also associated with deregulation in the vascular system, often combined with a defect in the immune system, as for diabetic foot ulcers and scleroderma [9, 10].

This highlights the need to add all these cells in models to better understand the mechanisms of wound healing to improve wound management. Animal models offer valuable insights but differ significantly from humans in immune responses and wound healing capacity across species [11, 12]. To overcome this issue, tissue engineering methods to reconstitute the skin 3D structure using human cells have been developed because of their ability to reproduce morphological and functional features of the native skin. Most of the 3D models consist of an epidermis and a dermis composed of fibroblasts embedded in a matrix [1]. They are used to study the re‐epithelialisation process and consist of the migration of epidermal cells into the wound bed to provide wound closure. Indeed, the epidermal closure is easily measurable and reflects in vivo mechanism [1, 13, 14, 15]. Although these models represent a significant advance in the field, the authors recognised certain limitations. Indeed, wound healing is not just an epithelial phenomenon since dermal closure requires the intervention of many cell types involved in inflammation, angiogenesis as well as the production and remodelling of the extracellular matrix. Crosstalk between cell types is essential, yet often missing. Furthermore, current models do not capture later healing stages and scar formation. These highlight the need for a more complex 3D model enabling the study of cell interactions and individual response variability.

Several strategies have emerged to introduce endothelial and immune cells to the skin models [1, 2, 3, 16, 17]. However, integrating those different cell systems remains a challenge in tissue engineering since each cell population is difficult to isolate and requires different culture conditions [18]. In addition, in most cases, the cells were isolated from different donors and their origin is not always skin‐related (e.g., immune cells from blood, or endothelial cells from umbilical cord), which limits the ability of these models to study specific characteristics of both individuals and the tissue as well as their response to treatment [19, 20].

Clinical observations reveal variability in skin structure and healing capacity among individuals. While pathological scarring is well documented, variability in the healing of healthy skin remains underexplored. Limited studies show differences in gene expression across clinically comparable skin samples, which may contribute to the diversity in response to injury [21]. A study carried out in humans showed that the reconstitution of the epithelial basement membrane after wounding of healthy skin is highly variable over time, ranging from 8 to 21 days for complete restitution [22]. Factors like age and ethnicity influence scar formation, but variability persists even within similar demographic groups. Not all patients develop hypertrophic scars, highlighting individual differences [23]. Similar findings apply to ulcer formation, where known risk factors don't fully explain outcomes [24]. Such variability limits the predictability and effectiveness of wound treatment.

Wound healing therapies consist of used bandages, drugs, devices, surgery, biological products to quickly restore skin integrity. Among biological therapies, platelet derivatives as platelet‐rich plasma (PRP) or platelet lysate (PL) are clinically used to improve healing and have proven their effectiveness on diabetic wounds [25, 26, 27]. A recent study in pigs demonstrated that the use of a PRP‐loaded artificial dermis promotes vascularisation and wound healing [28]. PRP is rich in growth factors and cytokines, the major ones being FGF, VEGF, and PDGF [29]. In wound healing, PRP could reduce lymphatic oedema [30] and accelerate the migration and proliferation of human fibroblasts and keratinocytes [31, 32] thus promoting matrix remodelling [33] and accelerating wound re‐epithelialisation. PRP also has an anti‐inflammatory effect by promoting macrophage polarisation towards the pro‐regenerative M2 phenotype [34].

To reproduce the complex interactions between the vascular system and the innate immune response provided by resident immune cells in human skin, we develop an autologous, vascularised, and immunocompetent human tissue‐engineered skin (aviTES) [35]. In this model, human primary cells isolated from the same patient are used to reconstitute a highly differentiated epidermis on top of a dermis containing different populations of immunocompetent cells (macrophages, lymphocytes, and dendritic cells) along with endothelial capillary networks and fibroblasts embedded in their own produced matrix. Thus, the study of resident immune cells offers the possibility of understanding the regulation of inflammatory phenomena in tissue, independently of circulating cells.

Since wound healing is linked to the immune and vascular systems and that genetic and epigenetic factors influence the phenotype of both cell types, we hypothesise that the aviTES model may reproduce inter‐individual variability to better understand the wound healing process. The aviTES were thus produced with cells from five different donors in two different laboratories. After wound induction, re‐epithelialisation across donors was quantified for each individual. Furthermore, different mechanisms involved in dermal healing, such as the ability of immune cells to migrate into wounds and the formation of the capillary network, were evaluated. We also treated the tissue‐engineered wounded skin with PL. For each studied parameter, individual variability was evaluated and linked to healing mechanisms.

## 2 Materials and Methods

### 2.1 Cell Extraction and Characterisation

Clinically healthy residual skin samples were collected from female patients aged from 21 to 58 undergoing reconstructive plastic surgery (Table S1). Experiments were carried out in two facilities, the LOEX Research Center (Quebec City, Canada) and the Silab's Research and Development Laboratory (Saint‐Viance, France) and followed the Declaration of Helsinki protocols. At LOEX, written informed consent was obtained from donors, and the study was approved by the Ethics Committee of the CHU de Quebec—Université Laval (#2017‐3591). At Silab, informed and oral consent was obtained according to the ethical guidelines set by the French law and with a dedicated authorisation for Silab's R&D team (no. DC‐2016‐2646, Cellule Bioéthique DGRI/A5, Paris, France). All the cutaneous cell populations used to reconstruct each skin model were derived from the same donor (autologous) and were cultured at similar passages (passage 2 for keratinocytes and fibroblasts). Epidermal and dermal cells were extracted as previously described [35]. Briefly, each skin biopsy was treated with dispase (Sigma‐Aldrich, Oakville, ON, CAN) to separate the dermis and the epidermis. The two skin compartments were then processed separately. Epidermis treatment with trypsin (Gibco, Grand Island, NY, USA) yielded keratinocytes which were then amplified by culture in Dulbecco's Modified Eagle's medium (DMEM Gibco, Waltham, MA, USA) with 3:1 Ham's F12 (Gibco), supplemented with 5% foetal calf serum (Wisent Inc.), 5 μg/mL of insulin (SAFC Bioscience, Lenexa, KS, USA), 0.4 μg/mL of hydrocortisone (Teva, Toronto, ON, Canada), 10 ng/mL of epidermal growth factor (R&D Systems, Oakville, ON, Canada), 0.212 mg/mL of isoproterenol hydrochloride (Sigma‐Aldrich, Oakville, ON, Canada), 100 IU/mL of penicillin (Fresenius Kabi, Homburg, Germany), and 25 μg/mL of gentamycin (Galenova, Saint‐Hyacinthe, QC, Canada). The dermis was treated with an enzymatic solution to obtain a mix of all dermal cells containing fibroblasts, endothelial cells, and immune cells. The mix of dermal cells was either frozen or cultured to obtain fibroblast populations in DMEM supplemented with 10% foetal bovine serum, 100 U/mL penicillin G, and 25 mg/mL gentamicin (Schering Inc., Pointe Claire, Canada). Flow cytometric analysis of the dermis extracted cells was performed independently at the two institutions, each using its own panel, as previously described [35] (Table S2).

### 2.2 Autologous, Vascularised and Immunocompetent Tissue Engineered Skin Model, aviTES

The aviTES model was carried out as previously described [35]. Experiments were conducted using cells from three donors collected at LOEX and two donors at Silab (Table S1). Briefly, fibroblasts were cultured using the self‐assembly approach to produce matrix and form two fibroblast sheets (3.8 cm^2^). After 28 days, amplified keratinocytes from the same donor were seeded on the top of one fibroblast sheet (named epidermal/dermal sheet) and cultured for an additional 4 days. Subsequently, the thawed mix of dermal cells, obtained from the dermis enzymatic digestion of the same donor, was embedded in a 1:1 purified type I bovine collagen solution (PureCol EZ gel solution, Sigma‐Aldrich) to prevent cell diffusion into the culture medium. This cell‐collagen mixture was spread on a dermal sheet and covered with the epidermal/dermal sheet to make the dermal‐epidermal construct. This step was followed by epidermal maturation at the air/liquid interface for 13 days in minimum medium (mDH) composed of Dulbecco's Modified Eagle's medium (Gibco, Waltham, MA, USA) with 3:1 Ham's F12 (Gibco) supplemented with 0.1% bovine serum albumin (Proliant, Ankeny, IA, USA), 0.1% foetal calf serum (Wisent Inc., St‐Bruno, QC, CAN), 100 U/mL penicillin G (Sigma‐Aldrich), and 25 μg/mL gentamycin (Gemini bio product, Sacramento, CA, USA).

### 2.3 Excisional Wounding of aviTES Models

After 13 days of culture at the air/liquid interface, a wound was made in the centre of the aviTES using a 2 mm diameter biopsy punch (Acuderm P250, Thermo Fisher Scientific, Waltman, MA, USA). The third fibroblast sheet composed solely of fibroblasts from the same donor and prepared as described above was added below the wounded aviTES to guide cell migration. The wounded aviTES was then placed at the air/liquid interface with 3 mL of mDH for the first 24 h to concentrate released cytokines. The conditioned media were collected at Day 1 and replaced with 7 mL of mDH. The wounded aviTES were maintained at the air/liquid interface for an additional 6 days with collections and renewal of the medium on Days 4 and 7. The collected conditioned media were stored at −80°C until analysis. To study the effect of PL (PLTmax Human Platelet Lysate, MilliporeSigma, St. Louis, MO, USA) on wound healing, culture media were supplemented with 5% PL starting from the time of wounding and at each medium change.

### 2.4 Re‐Epithelialisation Follow‐Up

Macroscopic photographs of the wounded aviTES were taken every day for 7 days on each aviTES using a ZEISS CL1500 Eco upright binocular microscope, V8 discovery with Axiocam ERc5s camera, 1.5× objective (achromat FWD28mm) (Carl Zeiss Meditec, AG, Oberkochen, Germany) at LOEX or with confocal ZEISS lsm 5, 5× objective lens at Silab. For each donor, three to five aviTES were generated per experiment and the wound closure area was measured using the area measurement tool of the Image J software (National Institutes of Health (NIH), Bethesda, USA). The closure area was then calculated using the following formula:

$$\%\text{of reepithelialisation}=100\left(1-\frac{\text{area}\;\mathrm{Day}\;x}{\text{area}\;\mathrm{Day}\;0}\right)$$

### 2.5 Histology

At Day 7 post‐wound, half of the wounded aviTES samples was harvested, cut in two parts and either fixed in 4% formalin (Thermo Fisher Scientific) for 24 h or embedded in Tissue‐Tek optimal cutting temperature (OCT) Compound (Sakura Finetek, Torrance, CA, USA), frozen in liquid nitrogen, and stored at −80°C. Fixed samples were dehydrated in successive alcohol baths, impregnated in xylene and embedded in paraffin using a Tissue‐Tek VIP6 (Sakura Finetek, Torrance, USA). Paraffin‐embedded samples were cut into 4 μm‐thick sections, then stained with Masson's trichrome (Weigert's haematoxylin, fuchsin‐ponceau and aniline blue) or haematoxylin, eosin, and saffron (DAKO Cover Stainer, Agilent, France). Epidermal thickness was measured in the injured (healed) region, including the stratum corneum. The average thickness was calculated by dividing the epidermal area by the length of the basal membrane, using Image J software.

### 2.6 Immunofluorescence

OCT‐embedded aviTES sample were cryosectioned at a thickness of 8 μm, permeabilised with 100% acetone for 10 min at −20°C and subsequently blocked with 5% goat serum for 20 min to prevent nonspecific antibody binding. The antibodies used are described in Tables S3 and S4, and cell nuclei were labelled with DAPI (Sigma‐Aldrich).

Whole mount fluorescence staining was performed on the remaining half‐aviTES samples. The aviTES samples were fixed for 2 h in 4% formalin and subsequently preserved in phosphate buffered saline (PBS). The aviTES samples were then incubated for 4 h with a blocking and permeabilisation buffer (PBS: 100 mM pH 7.4, NaCl 150 mM + triton 0.5% + goat serum 5%). Primary antibodies diluted in PBS were incubated overnight at 4°C. Six 20‐min rinses were performed at room temperature in PBS + tween 0.05%. Secondary antibodies were incubated for 2 h. The antibodies used are described in Tables S3 and S4. Images were taken using the Zeiss LSM700 confocal microscope equipped with an AxioCam HR Rev3 camera (Carl Zeiss Meditec) at LOEX or the Axio‐Observer Z1 confocal (ZEISS) at Silab.

### 2.7 Quantification of IL‐8, Pro‐Collagen‐I and MMP Secretion

ELISA kits were used to assess IL‐8 and pro‐Collagen‐I release (Duoset DY208 and DY6220 respectively, R&D system). A 1:60 and a 1:2400 dilution were required for IL‐8 and pro‐collagen‐1 assays, respectively. The ELISA reaction was measured at 450 nm using a SpectraMax Plus 384 microplate spectrophotometer (Molecular Devices, San Jose, CA, USA). The activity of MMP was measured using a fluoroenzymatic assay kit (SensoLyteTM 520 Generic MMP assay kit, Anaspec, Fremont, CA, USA) and a SpectraMax ID3 multi‐mode microplate reader (Molecular Devices). Since the MMP kit includes a 4‐aminophenylmercuric acetate (APMA) activation step and subsequently detects the enzymatic activity of MMP‐1, −2, −7, −8, −9, −12, −13, and −14, the results reflect the overall secretion of MMPs present in the culture medium. All procedures were carried out at room temperature, following the manufacturer's instructions, and samples were tested in 3–5 technical replicates. PL was assayed, and its contribution was subtracted from the final results. Notably, PL did not contain procollagen and showed only minimal levels of MMP.

### 2.8 Statistical Analysis

Independent *t*‐tests with the two‐stage step‐up method of Benjamini, Krieger and Yekutieli were performed using GraphPad Prism Software 8 (San Diego, CA, USA). Model repeatability was assessed by calculating the relative standard deviation (RSD) of wound area measurements between technical replicates of control conditions within the same experiment. This calculation was made for all five donors. Model reproducibility was defined by comparing the percentage of wound closure between experiments using cells from the same donor. For ELISA and fluoroenzymatic assay data, a two‐way ANOVA followed by multiple unpaired *t*‐tests was used to compare secretion levels on each day post‐injury. All values are presented as mean value ± standard deviation (SD). The significance threshold was set at 0.05.

## 3 Results

### 3.1 Skin Wound Healing Model Validation

Two different flow cytometry panels were used to characterise each donor's cell extraction as previously described [35]. The proportions between the different cell types were of the same order of magnitude across donors (Table S1). Lymphatic microvascular endothelial cells (LECs), blood microvascular endothelial cells (BECs), immune cells, and fibroblasts were present in each extraction. Immune cells accounted for 26.3% ± 6.4% of cells, while 10.1% ± 3.3% were endothelial cells (LECs and BECs), with the remaining cells being fibroblasts/fibrocytes. However, individual variability was notable, particularly in the percentage of endothelial cells (from 2.8% to 11.3% for BECs and from 1.5% to 3.9% for LECs).

In the unwounded region of the aviTES, endothelial cells spontaneously formed blood and lymphatic capillary networks, discriminated by varying levels of CD31 protein expression on endothelial cells. BECs exhibited high CD31 levels, whereas LECs showed lower CD31 expression and selectively expressed the lymphatic‐specific marker LYVE‐1 [36, 37] although expression was not uniform across the LEC population. In addition, lymphatic capillaries had a larger overall diameter than blood capillaries as noted in the literature [38]. Capillary density varied between donors and no lymphatic vessels were detectable in aviTES from donors D and E (Figures S1 and 3). Immune cells were evenly distributed throughout the dermis in all aviTES. Macrophages or dendritic cells, labelled with the CD206 antibody, were observed near the capillaries and adopted a star shape with dendrite extensions (Figure S1).

#### 3.1.1 Reproducibility of Re‐Epithelialisation of the Wounded aviTES

**FIGURE 1:**
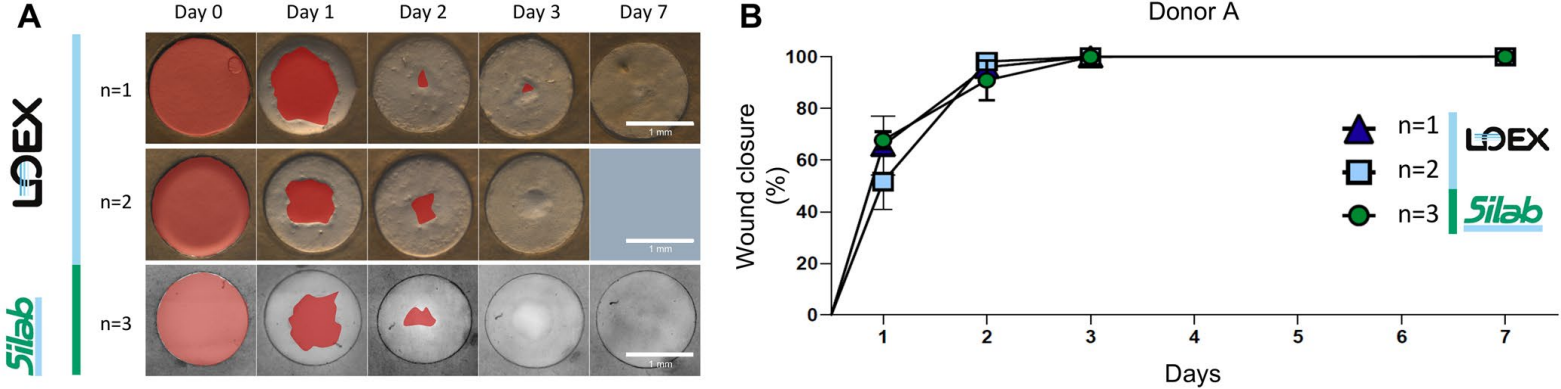
Reproducibility of re‐epithelialisation in the aviTES wound healing model. Re‐epithelialisation of autologous vascularised and immunocompetent tissue engineered skin, aviTES, reconstructed with donor A cells in two laboratories (LOEX and Silab). (A) Macroscopic photographs of the wounded aviTES taken over the days until complete re‐epithelialisation. The red areas represented the part of the wound that was not closed. Scale: 1 mm. Three 5 technical replicates/experiment, *n* = 3 independent experiments. (B) Graphical representation of the percentage ± SD of wound closure of aviTES produced with donor A cells.

To further assess repeatability and reproducibility of the method, the wound healing model was performed three times in two independent laboratories using aviTES constructs derived from cells isolated from the same donor (donor A). Two‐millimetre diameter wounds were made in the aviTES, and wound re‐epithelialisation was monitored over time (Figure 1). In all cases, complete re‐epithelialisation was observed within 3 days (Figure 1A). For each experiment (comprising 3–5 technical replicates), a relative standard deviation (RSD) was calculated allowing the calculation of a mean RSD of 7.9%, with a standard deviation of ±2.7% across the three experiments. This low intra‐experimental variability demonstrates strong repeatability, while the consistent results across laboratories confirm the model's robust reproducibility (Figure 1B).

**FIGURE 2:**
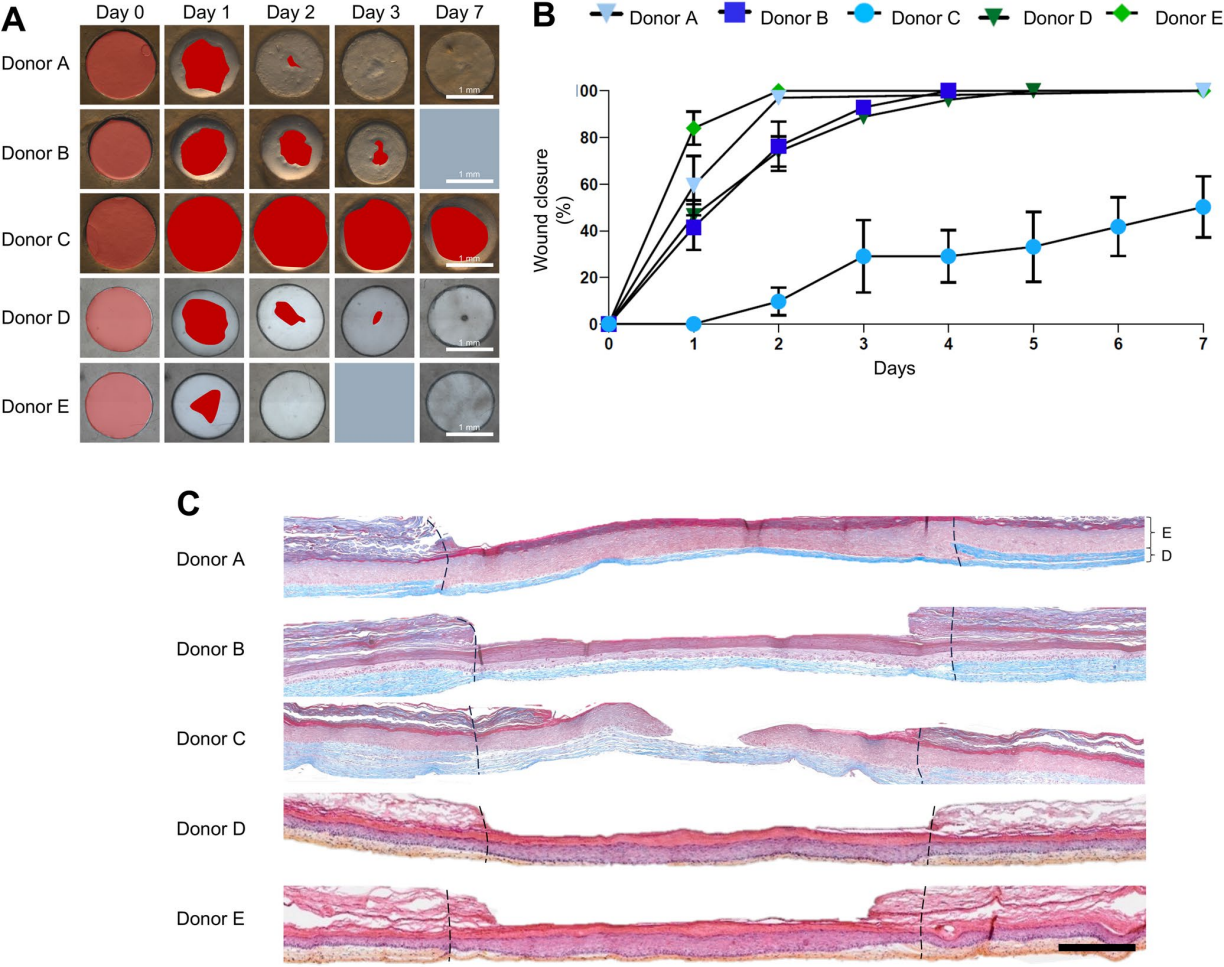
Variability of re‐epithelialisation between donors. (A) Macroscopic wound re‐epithelialisation of autologous vascularised and immunocompetent tissue engineered skin, aviTES using cells from five different donors. The red areas represented the part of the wound that was not closed. Scale: 1 mm. (B) Graphical representation of wound closure ±SD of reconstructed aviTES skins from five donors. Three to five technical replicates/experiment, *n* = 1–3 independent experiments. (C) Histology with Masson trichrome (donors A–C) or haematoxylin eosin saffron (donors D‐E) staining of the wound areas 7 days post‐injury. Wound margins are indicated by dashed black lines. E: Epidermis, D: Dermis. Scale: 500 μm.

Cells from four additional donors have been used to reconstruct the aviTES wound healing model. Variability in the re‐epithelialisation rate was observed between the different donors. In most cases, complete re‐epithelialisation was observed within 2–4 days except for aviTES reconstructed with cells isolated from donor C skin that were not healed after 7 days (Figure 2A,B). Histological analysis of aviTES 7 days post‐wounding revealed the presence of well‐differentiated stratified epidermis in the wound area for all donors, with a thickness ranging from 152.07 ± 7.99 to 215.18 ± 4.66 μm (Table S5). Except for donor C, the epidermis completely covered the wound, confirming successful re‐epithelialisation (Figure 2C). The mean RSD of the re‐epithelialisation percentage between replicates for each donor was 7.05% ± 4.13% confirming the repeatability regardless of the donor and the laboratory in which the experiments were conducted (Figure 2B).

To explore the reasons for the different rates of closure, growth, and migration capacities of keratinocytes and fibroblasts from donors A–C were evaluated (Figures S2 and S3). Cells exhibited exponential growth between 24 and 96 h post‐seeding, after which they transitioned into a stationary phase (Figure S2A,C). Calculated doubling times for keratinocytes and fibroblasts revealed variability between donors. Keratinocytes from donor C displayed a significantly faster growth rate, with a doubling time approximately 7 h shorter than that of the other donors, followed by donor B and then donor A (Figure S2B). For fibroblasts, donors A and B showed comparable growth rates, while fibroblasts from donor C exhibited a slightly slower growth rate (Figure S2D). The study of the migration capacity of keratinocytes showed that the capacity of cells from donor C was significantly lower than that of cells from donors A and B (Figure S3A,B). In contrast, fibroblast migration capacity was similar across all donors (Figure S3C,D).

#### 3.1.2 Endothelial and Immune Cell Migration Into the Wound

**FIGURE 3:**
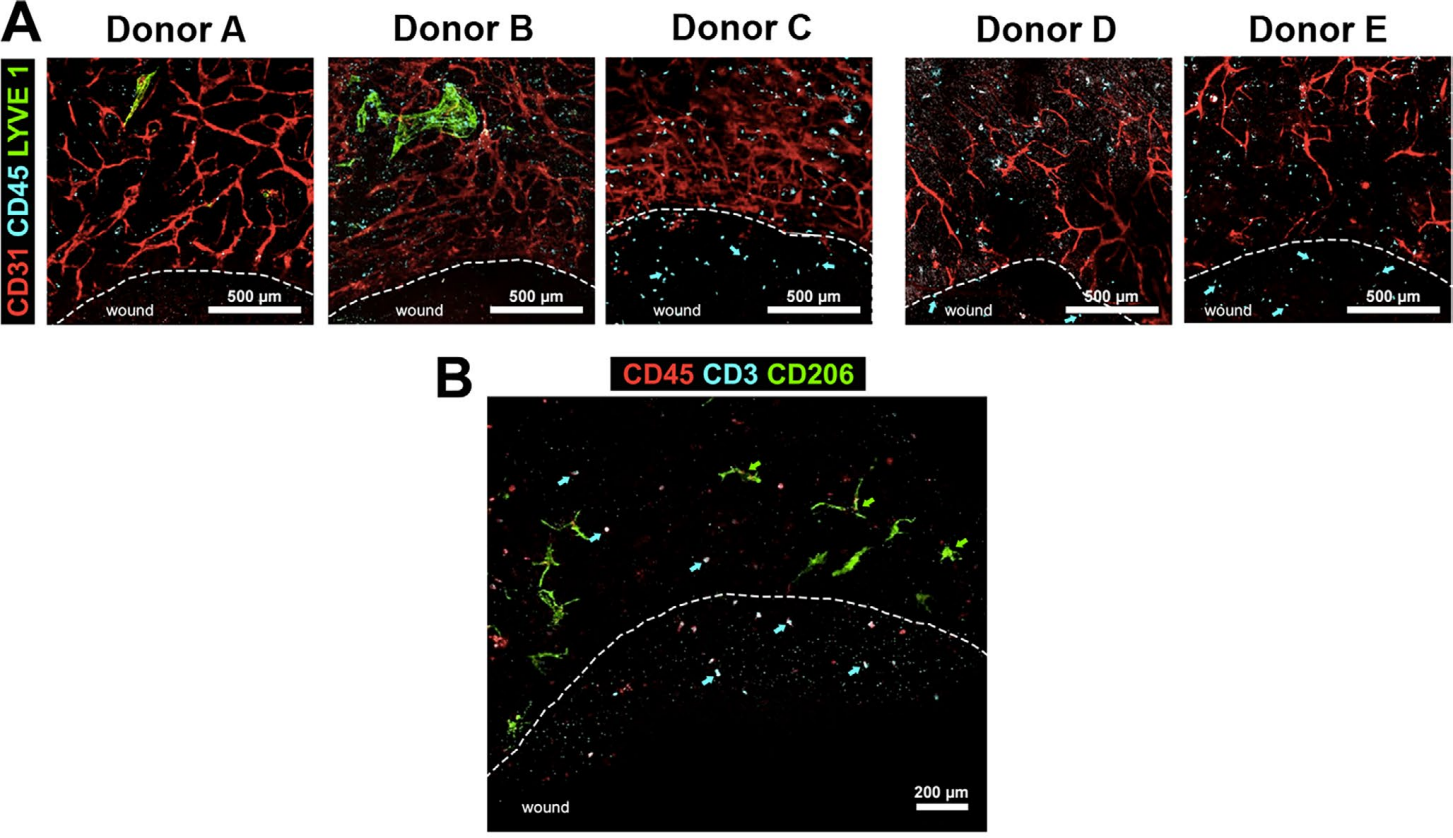
Migration of immune cells into the wound. Whole‐mount immunofluorescence analysis of the wounded aviTES 7 days post‐wounding. (A) Blood microvascular endothelial cells (CD31+ high, red) and lymphatic microvascular endothelial cells (CD31+ low, LYVE‐1+, predominantly green) remained confined to unwounded regions and did not migrate into the wound area. In contrast, immune cells (CD45+, blue) migrated into the wound site. Dashed lines indicate the wound margins. Data represent results from two independent experiments per donor. Scale bars: 500 μm. (B) Lymphocytes (CD45+/CD3+, blue and red, highlighted with blue arrows) were the main immune cells to migrate into the wound area. Myeloid cells (CD45+/CD206+, green and red, highlighted with green arrows) and other immune cells (CD45+, red) were restricted to the unwounded part of the aviTES. The dashed line delimits the wound margins with the “wound” indicating the inner part of the wound. The image was produced using cells from donor A and is representative of donors A–C. Scale bar: 200 μm.

Angiogenesis is a process that occurs during wound healing, following the inflammation phase. Seven days after wounding, endothelial cell migration into the wound was not observed following double CD31/LYVE‐1 labelling (Figure 3A). Immune cell migration into the wound site represents an early and critical step in the physiological process of human wound healing. At 7 days post‐wounding, immune cells (CD45+), which were absent from the dermal sheet placed beneath the wound, were observed within the wound area in aviTES models generated from four of the five donors (Figure 3). Most of the immune cells detected in the wound area were identified as T lymphocytes (CD45+/CD3+, Figure 3B). A smaller population of CD45+/CD3− cells was also observed and could be B lymphocytes or innate lymphoid cells (Figure 3B). Myeloid cells (CD206+) were observed exclusively in the unwounded dermis but not in the wound bed (Figure 3B).

#### 3.1.3 Production of Cytokines During Healing

**FIGURE 4:**
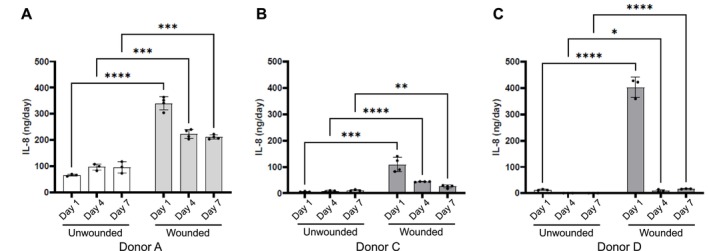
IL‐8 secretion induced by injury and during the wound‐healing process over 7 days. The secretion of IL‐8 was assessed by ELISA in the conditioned media of aviTES without (white bars) or following wound formation (grey bars). (A) and (B) represent results obtained with donors from the LOEX, while (C) was obtained with a donor from Silab. Results are expressed in ng/day ± SD. Two‐way ANOVA followed by multiple unpaired *t*‐test comparing each time point. ****p ≤ 0.0001; ***p ≤ 0.001; **p ≤ 0.01; *p ≤ 0.05.

One of the main mechanisms orchestrating the healing process is the secretion of soluble mediators known as cytokines. To assess whether it is possible to quantify these stimuli in the model, IL‐8 (CXCL8), one of the main inflammatory cytokines involved in the first stage of healing [39], was measured in the injured and uninjured aviTES produced using cells from 3 different donors. In culture medium from uninjured skin, IL‐8 was weakly and consistently detected, with variations depending on the donor (Figure 4). Notably, donor A exhibited a higher basal IL‐8 secretion. After wounding, a substantial release of IL‐8 was observed in the conditioned media of injured aviTES skin during the first 24 h post‐wounding, followed by a decrease in IL‐8 secretion from Day 4 onwards, returning almost to basal levels (Figure 4). Interestingly, donor C showed the lowest IL‐8 secretion post‐injury, even though the basal level in uninjured skin was comparable to that of donor D.

### 3.2 Influence of a Pro‐Regenerative Environment on the Reconstructed Skin Model

To compare and validate the response of reconstructed skins to an in vivo wound healing stimulus, 5% PL was added to the culture medium on the day of wound formation. We used this well‐known proregenerative treatment to compare the response of reconstructed skins with the literature on wound re–epithelialisation, endothelial, and immune cell migration.

#### 3.2.1 PL Tended to Accelerate Re‐Epithelialisation

**FIGURE 5:**
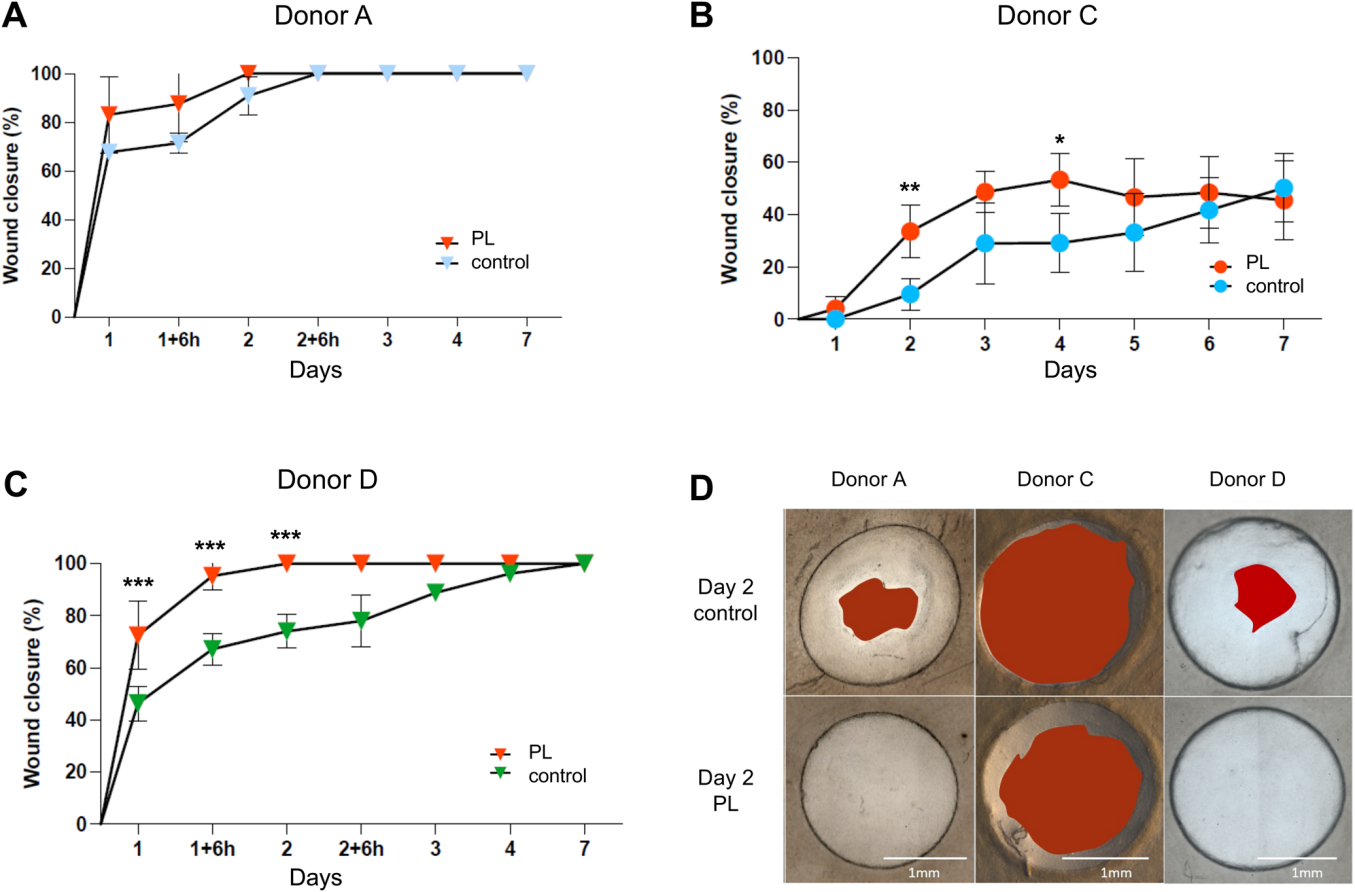
Influence of platelet lysate (PL) treatment on the re‐epithelialisation of wounded autologous vascularised and immunocompetent tissue engineered skin, aviTES. (A) Donor A and (B) donor C were from LOEX while (C) donor D was from Silab. Graphical representation of the re‐epithelialisation ±SD over time from Day 0 and for each donor with or without PL in the culture medium. *N* = 3 donors, 3–5 technical replicates/donors. Multiple unpaired *t*‐test per donor. ***p ≤ 0.001; **p ≤ 0.01; *p ≤ 0.05. (D) Macroscopic images of wounded skins at Day 2 with or without PL treatment using cells from three different donors. The red areas represented the part of the wound that was not closed. Scale: 1 mm.

Wounded aviTES were treated with PL (5%) for 7 days and re‐epithelialisation was macroscopically monitored throughout the treatment period. From Day 1, an increase in the rate of re‐epithelialisation was observed when PL was added (Figure 5). With PL treatment, the wound was fully closed after 2 days in aviTES using cells from donors A and D, while only an improvement was visible for aviTES from donor C compared with the control condition (Figure 5). However, despite PL treatment, the wound of donor C failed to achieve complete closure by Day 7 (Figure 5B,D).

#### 3.2.2 PL Improved the Capillary Network Structure and Neo‐Angiogenesis

**FIGURE 6:**
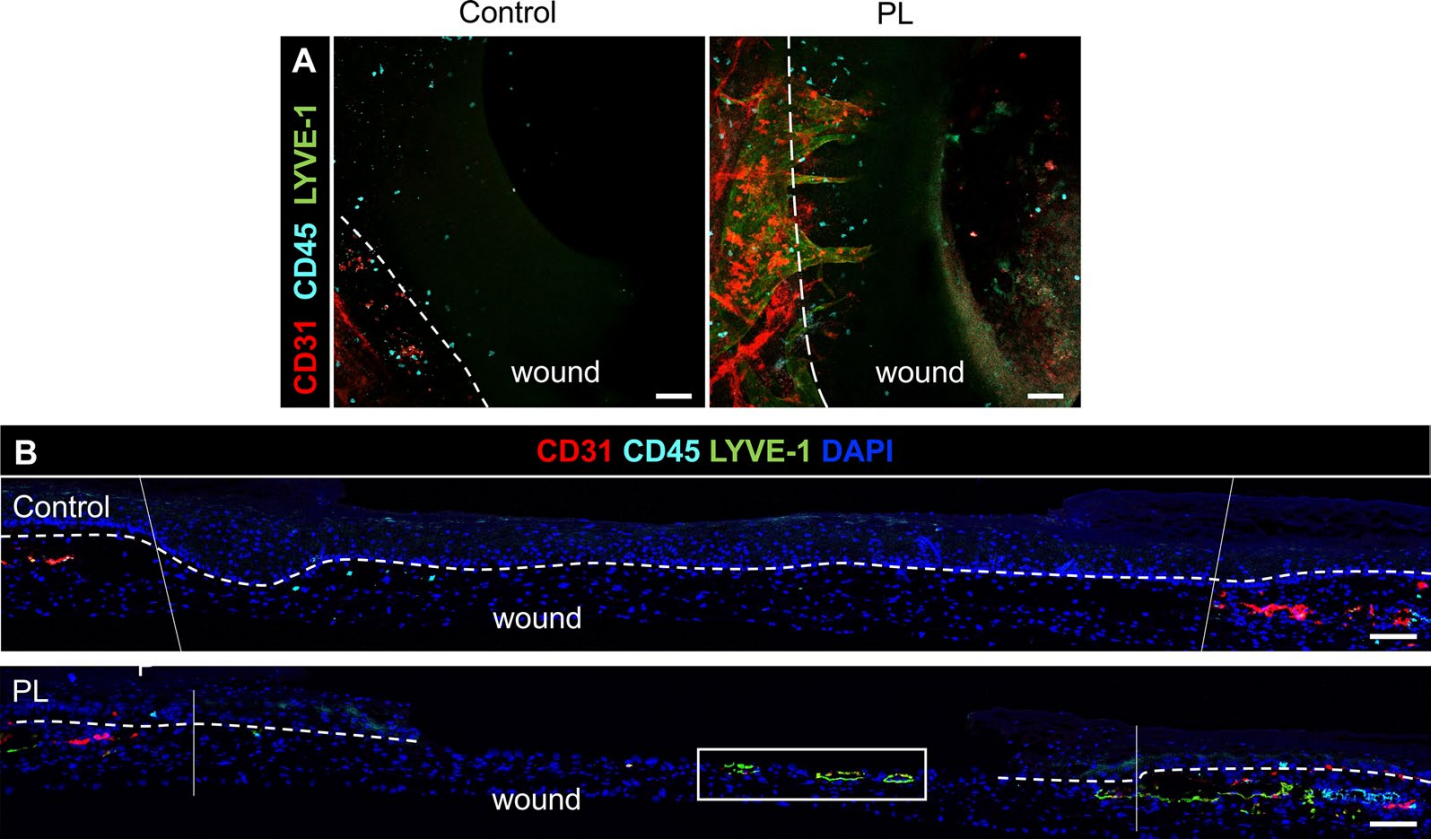
Effect of platelet lysate (PL) on endothelial and immune cell migration from the edges into the wound. Immunofluorescence of the wounded aviTES reconstructed with cells from donor C was performed 7 days post‐wounding with or without PL treatment with 2–3 technical replicates. While only immune cells (CD45+, light blue) migrated into the wound in the control condition, blood microvascular endothelial cells (CD31+ high, red) and lymphatic microvascular endothelial cells (CD31+ low/LYVE‐1+, predominantly green) invaded the wound bed in the presence of PL. (A) 3D whole mount microscopy of the wound merge. Scale bar 100 μm. The dashed line delimits the wound margins. (B) Transversal section of the wound. Scale bar 200 μm. The inset shows the presence of lymphatic vessels (CD31+/LYVE‐1+, predominantly green) within the wound bed. The dashed lines indicate the demarcation between the epidermis and dermis. Dark blue: DAPI staining.

At 7 days post‐wounding, immunofluorescence staining of wounded aviTES with or without PL was performed to evaluate its influence on cell migration and skin architecture. In the unwounded parts of aviTES treated with PL, we observed a trend towards increased LYVE‐1+ lymphatic capillary density (donors A and C, Figure S4). In the aviTES reconstructed with donor D cells, no lymphatic network has been detected but a slight increase in CD31+ blood vessel density was observed following PL treatment (Figure S4C,F). In the wound, endothelial cell migration was absent in the control samples but was observed in the wound bed of models stimulated with PL and produced using donor C cells (Figure 6). PL treatment did not affect immune cell migration. No myeloid cells were observed in the wound and no increase in the density of lymphoid cells was observed in the PL treated condition (Figure 6).

#### 3.2.3 Pro‐Collagen‐1 and Matrix Metalloproteinase Secretion in Wounded aviTES Varied Over Time

**FIGURE 7:**
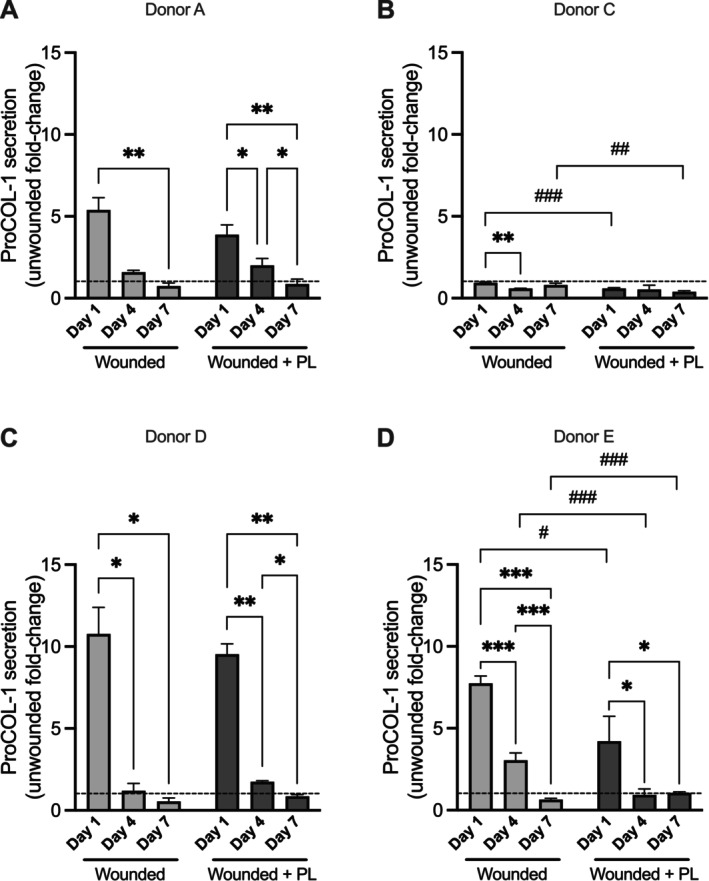
Secretion of pro‐collagen I in conditioned media of aviTES models. Secretion of pro‐collagen I was assessed using ELISA in the conditioned media of unwounded aviTES and wounded aviTES model treated without (light grey bars) or with PL (dark grey bars) over a 7‐day period. Data represent results in ng/day ± SD from four donors with 3–5 technical replicates/donor. Two‐way ANOVA. ****p ≤ 0.0001; ***p ≤ 0.001; **p ≤ 0.01; *p ≤ 0.05. PL‐specific effects are marked as ^###^ *p* ≤ 0.001; ^##^ *p* ≤ 0.01; ^#^ *p* ≤ 0.05.

**FIGURE 8:**
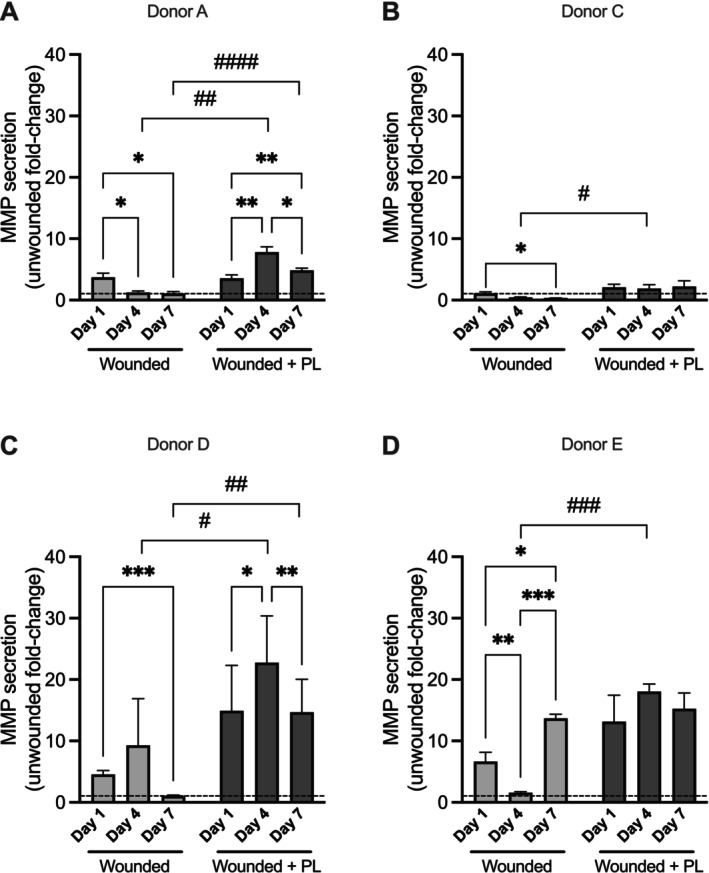
MMP secretion in conditioned media of aviTES models. MMP secretion was measured using a fluoroenzymatic assay in conditioned media collected from unwounded aviTES and wounded aviTES treated without (light grey bars) or with PL (dark grey bars) over a 7‐day period. Results are fold‐change ±SD relative to unwounded aviTES from four donors, with 3–5 technical replicates/donor. Two‐way ANOVA; ****p ≤ 0.0001; ***p ≤ 0.001; **p ≤ 0.01; *p ≤ 0.05 (day‐to‐day comparison). PL‐specific effects are marked as ^###^ *p* ≤ 0.001; ^##^ *p* ≤ 0.01; ^#^ *p* ≤ 0.05.

Since matrix remodelling is an essential step in wound healing, collagen I (Figure 7) and matrix metalloproteinase (MMP) secretion (Figure 8) were assessed in the culture media of uninjured and injured aviTES, with or without PL treatment. Except for donor C, pro‐collagen I secretion followed a similar general pattern, but with different levels of secretion depending on the cell donor (Figure 7). Following injury, pro‐collagen I production significantly increased in the conditioned medium of aviTES using cells from donors A, D, and E, whereas a decrease was observed in donor C aviTES. This may be linked to the basal level of pro‐collagen secretion by unwounded aviTES, which was very high when donor C cells were used (8551.2 ± 1892.8 ng/day) compared with other donor cells (< 1000 ng/day). Over time, pro‐collagen I secretion decreased, returning to levels similar to those observed in uninjured aviTES by Day 7. PL treatment slightly decreased collagen I secretion at Day 1, but no significant effects were observed thereafter (Figure 7).

At Day 1 after wounding, MMP secretion by aviTES increased by less than fivefold compared to unwounded controls, irrespective of the cell donors, except for donor C, who exhibited minimal changes. In contrast, in the presence of PL, MMP secretion significantly increased by 10–20‐fold, particularly at Day 4 post‐wounding. The level of secretion varied depending on the cell donor, except for cells from donor C, which showed a marginal increase in MMP secretion.

## 4 Discussion

Current tissue engineered skin healing models fail to reproduce the complex interactions between vascular, immune, and cutaneous cells due to challenges associated with co‐culture of these cell types and their immune compatibility [18, 40]. However, the representation of these systems is essential to reproduce the complete wound healing process [3, 41]. Furthermore, the variability of individual responses to treatments, leading to correct or pathological healing outcomes, remains poorly understood. In the present study, the autologous, vascularised, and immunocompetent Tissue‐Engineered Skin (aviTES) was used to analyse the wound healing process in vitro. The model was replicated using cells from five donors in two independent laboratories located in two different countries to assess its technical reproducibility and to evaluate whether interindividual variability could be observed. In addition, plasma lysate, a plasma rich platelet‐derived product known to enhance wound healing in vivo, was tested to validate the similarity of the model's response to the native human skin.

The aviTES model was successfully reproduced in both laboratories. Although different antibodies and cell sorting methods were employed, the resulting cell proportions after dermal cell extraction were consistent across donors and aligned with previously published data [35]. These findings demonstrate the reproducibility of our extraction methodology. In addition, the results showed that, for the same donor, it was possible to obtain very similar wound closure rates across laboratories, demonstrating the robustness of this in vitro wound healing model. Subsequently, cell populations isolated from five donors were analysed to assess the feasibility of studying inter‐donor variability. Overall, the aviTES model faithfully reproduces the natural diversity that exists between individuals, with the speed of re‐epithelialisation and capillary density varying from one donor to another.

The skin wound closure depends on multiple parameters, such as the wound size or the age of the donor [42]. To our knowledge, only one study has assessed the rate of reepithelialisation in vivo on healthy human skin. The estimated healing rate reported in this study was less than 0.1 mm per day [43]. Human tissue engineering models based on exogenous rat‐tail collagen showed a complete re‐epithelialisation of a 3 mm wound in 4 days [15]. Smaller wounds on ex vivo excisional models (2 mm) closed within 4–5 days [44]. Other similar ex vivo models reported a complete closure by Day 7 [45], as well as in the tissue engineering model of Laplante's study [13]. This discrepancy may be due to the model used and the environmental conditions but also to donor epigenetic variability. In the wounded aviTES model, we observed complete re‐epithelialisation of a 2 mm diameter wound in 2–4 days but in one case, the healing rate was significantly lower. It is difficult to determine the cause of the discrepancy observed between donors, which may be attributed either to differences in culture conditions or to donor‐specific factors. No differences were observed in donor parameters, including age, sex, biopsy site, cellular distribution and count within the biopsy, as well as in the methodologies used for extraction and culture. However, unknown health conditions (such as diabetes) may contribute to the observed discrepancy. The impact of diabetes or other pathologies on cellular response in our model will be investigated in future experiments. To further investigate the delay in epidermal closure, we analysed intrinsic cellular parameters, including proliferation or migration rate. If proliferation cannot explain the discrepancy observed with the aviTES model, re‐epithelialisation may be linked to keratinocyte migration capacity, highlighting the fact that this parameter is more important during re‐epithelialisation than keratinocyte growth capacity.

In this model, we created an excision wound to provide a sufficiently large surface to re‐epithelialise on a cellular scale, and for microscopic changes to be perceived and studied. This wound model mimics the effect of a mechanical injury, and we were able to measure the cellular and local response to the wound, a response often masked by blood coagulation and the massive influx of blood cells into the wound. For example, at Day 1, an increase of IL‐8 cytokines was detected showing the cells' response to injury. Macrophages, endothelial cells, and epithelial cells produce IL‐8 in response to tissue injury, triggering the inflammatory reaction [39, 46, 47]. The observed increase of IL‐8 secretion may result from activation of keratinocyte TLR‐3 receptors by the double‐stranded RNA from damaged cells [48]. The increased secretion of pro‐collagen I and MMPs in the first day's post‐wounding confirmed the rapid activation of skin cells in response to changes in the local environment due to injury. Moreover, for each of these parameters, even if the observed changes followed a similar pattern, variability in secretion level was noted. That may reflect the individual variability and could explain the different cell response to the wound formation.

Environmental conditions, including low concentration of growth factors, existing during aviTES production were sufficient to support epidermal differentiation and capillary network organisation. After tissue perturbation by wounding, epidermal cells grow and migrate to reconstitute neo‐epidermis, but environmental conditions were not sufficient to stimulate neo‐angiogenesis. The absence of neovascularisation is unlikely to be due to a lack of endothelial cells as our previous work demonstrated the presence of pre‐formed vessel‐like structures in the tissue prior to injury [35]. Moreover, the addition of PL stimulated neo‐angiogenesis, demonstrating the ability of endothelial cells to respond to pro‐angiogenic signals. This model may thus be very helpful for better understanding angiogenic mechanisms and the role of drugs or cytokines. Optimisation of culture conditions with pro‐angiogenic factors could also lead to complete vascularisation as it was observed in a 3D dermal wound‐on‐chip model [49] or in ex vivo culture models [44].

The inflammation phase is mainly linked to the massive influx of blood cells into the wound. However, the resident immune role in wound healing is only beginning to be studied [50, 51]. Adding immune cells increases the relevance of skin models, especially to study healing mechanisms. For example, macrophages are central to the wound healing process, as depletion of these cells prevents complete in vivo healing [52]. Those cells were previously added to healthy and pathological skin models and spontaneously adopted a pro‐regenerative phenotype in healthy and fibrotic skin, and a pro‐inflammatory phenotype in diabetic models [53, 54]. Kreimendahl's skin model integrated blood monocytes into a 3D vascularised skin model. These cells were able to differentiate into macrophages, adopt a pro‐healing phenotype, migrate into the skin, and influence vasculature [55].

In the aviTES model, CD206+ cells could be associated with a pro‐regenerative macrophage phenotype [56]. The fact that they did not migrate into the wound confirms previous findings that resident macrophages have a lower migration capacity than monocyte‐derived macrophages [4]. In contrast to macrophages, high numbers of T lymphocytes (CD3+) were observed in the wound bed 7 days post‐wounding. Since no specific chemoattractant was present in the wound area, the observed migration likely reflects a non‐specific movement of cells. Regulatory T cells are implicated in skin regeneration by secreting anti‐inflammatory cytokines and promoting re‐epithelialisation [57]. As immunofluorescence labelling was performed after total re‐epithelialisation, we were unable to assess whether lymphocytes migrated before or after epidermal migration. Sequential sampling could be performed to better understand the migration mechanisms of these cells.

PL is rich in growth factors produced following platelet activation and used for its pro‐healing capacities to treat chronic wounds [27, 58]. In the aviTES model, treatment of the wound with PL demonstrated an improvement in epidermal and dermal wound healing. An accelerated rate of re‐epithelialisation, neo‐angiogenesis and an increase in vascular density were observed. PL contains numerous growth factors promoting the growth of keratinocytes and fibroblasts that could explain the faster wound closure [29]. PL also contains VEGF, which may explain the observed effects on endothelial cells [59]. The results obtained with PL are therefore in line with those obtained in vivo with this treatment. Moreover, despite the general improvement, a variable response was observed depending on the origin of the cells (donor). This underlines the interest of aviTES as a model reflecting individual variability in order to better understand wound healing mechanisms and test new drugs. Although more labour‐intensive, this model offers better reproducibility and longer viability than ex vivo explants. It allows for standardised conditions, customisable features, and is more ethically and logistically sustainable. These advantages make aviTES a more robust and versatile platform for research and testing.

All phases of wound healing are regulated by cellular players in the dermis and epidermis, interacting through the cutaneous, vascular, and immune systems. In addition to epidermal closure, the inclusion of dermal elements to recreate all these systems will enable the study of the underlying mechanisms of wound healing in depth as well as individual variability. In such a context, the use of human 3D skin models becomes imperative. The aviTES models provide a powerful system that closely mimics human physiology, enabling the evaluation and development of effective therapeutic approaches to wound healing, paving the way for advances in personalised medicine.

## Author Contributions

Emilie Attiogbe, Elodie Mareux and Sébastien Larochelle designed the study, conducted the experiments, and analysed the of data. Emilie Attiogbe, Elodie Mareux and Véronique J. Moulin wrote the manuscript. Caroline Gilbert and Véronique J. Moulin participated in the conception and interpretation of data and critically revised the manuscript. Carine Mainzer, Adèle Mauroux, Sandrine Gofflo, and Sylvie Bordes contributed to the study conception and revision of the manuscript. Brigitte Closs participated in the interpretation of data and all the authors gave their approval of the submitted version.

## Funding

This work was supported by Silab (France) and MITACS. Emilie Attiogbe received studentships from MITACS (IT12748) and from the Centre de recherche en Organogénèse Expériemental de l'Université Laval/LOEX. We acknowledge the support of the CHU de Québec—Université Laval Research Center supported by the Fonds de recherche du Québec—Santé (FRQS) and the Quebec Network for Cell, Tissue, and Gene Therapy—ThéCell (a thematic network supported by the Fonds de recherche du Québec–Santé).

## Conflicts of Interest

The authors declare no conflicts of interest.

## Supporting information


**Table S1:** Flow cytometry characterisation of dermal cells according to donors. Dermal cell proportion following extraction for each donor by flow cytometry characterisation made at the LOEX Research Center (Quebec City, Canada) and at the Silab's Research and Development Laboratory (Saint‐Viance, France). BEC, blood endothelial cells; LEC, lymphatic endothelial cells. Results are expressed in percentage of total extracted dermal cells. Age in years.
**Table S2:** Antibodies used for cell characterisation using flow cytometry.
**Table S3:** Primary antibodies used for cell characterisation using immunofluorescence method.
**Table S4:** Secondary antibodies from Invitrogen used for cell characterisation using immunofluorescence method.
**Table S5:** Epidermal thickness of aviTES 7‐day post‐wounding measured using Masson trichrome (donors A–C) or haematoxylin eosin saffron (donors D–E) staining.
**Figure S1:** Whole‐mount immunofluorescence analysis of the unwounded aviTES. Immune cells (CD45+, blue; indicated by blue arrows in donor B) were evenly distributed. Lymphatic endothelial cells formed larger capillaries (CD31+ low/LYVE‐1+, predominantly green in donor B and red in donor C, delimited with dashed lines) compared to blood capillaries (CD31+ high, red, indicated by red arrows). Macrophages or dendritic cells (CD206+, green in donor C) were observed near the capillaries, exhibiting a star‐like morphology with extensions. Scale bar: 100 μm. Images are representative of donors A–C.
**Figure S2:** Growth of keratinocytes and fibroblasts from donors A–C cultured on plastic through 6 days. Growth curves show exponential growth in A. keratinocytes and C. fibroblasts. After 96 h, the cells have entered stationary phase. Doubling time calculations of B. keratinocytes and D. fibroblasts revealed slight significant differences in growth rates between donors. Statistical significance was assessed using one‐way ANOVA: *p < 0.05; **p < 0.01; ***p < 0.001. *N* = 3 donors, with three technical replicate/donor and two independent experiments.
**Figure S3:** Migration of keratinocytes and fibroblasts from donors A–C cultured in DH minimum. (A) Keratinocytes and (C) fibroblasts were grown until confluence then scraped with a 200 μL pipette tip. Cells were washed with PBS and replaced by DH minimum. Pictures were taken right after the scratch (T0) and at 6 (T6), 18 (T18) and 24 h (T24). Scale bar: 200 μm. The migration rate of (B). Keratinocytes and (D) fibroblasts is represented as the percentage of the wound area. *N* = 3 donors with three technical replicate/donor and two independent experiments. The black dotted lines were included to represent the calculated slope of wound area reduction. The slope for keratinocytes from donor C was statistically different from the other donors.
**Figure S4:** Influence of platelet lysate (PL) treatment on blood and lymphatic networks in aviTES models. Blood endothelial cells (CD31+ high, red) and lymphatic endothelial cells (CD31+ low/LYVE‐1+, predominantly green) networks were visualised in the unwounded areas of the aviTES reconstructed with cells from 3 donors and cultured for 7 days either without PL (A–C) or with PL (D–F). Images are representative of 2–3 technical replicates/donors. Scale: 100 μm.

## Data Availability

The data that support the findings of this study are available on request from the corresponding author. The data are not publicly available due to privacy or ethical restrictions.
