# Supplementary material for: An Autologous, Vascularized and Immunocompetent Tissue Engineered Skin to Highlight Inter‐Individual Variability to Better Understand the Human Wound Healing
Source: Wound Repair Regen. 2026 Jan 29;34(1):e70129. doi: 10.1111/wrr.70129 (PMC12854395; doi:10.1111/wrr.70129)
Supplement: Supplementary file 1 — Table S1: Flow cytometry characterisation of dermal cells according to donors. Dermal cell proportion following extraction for each donor by flow cytometry characterisation made at the LOEX Research Center (Quebec City, Canada) and at the Silab's Research and Development Laboratory (Saint‐Viance, France). BEC, blood endothelial cells; LEC, lymphatic endothelial cells. Results are expressed in percentage of total extracted dermal cells. Age in years. Table S2: Antibodies used for cell characterisation using flow cytometry. Table S3: Primary antibodies used for cell characterisation using immunofluorescence method. Table S4: Secondary antibodies from Invitrogen used for cell characterisation using immunofluorescence method. Table S5: Epidermal thickness of aviTES 7‐day post‐wounding measured using Masson trichrome (donors A–C) or haematoxylin eosin saffron (donors D–E) staining. Figure S1: Whole‐mount immunofluorescence analysis of the unwounded aviTES. Immune cells (CD45+, blue; indicated by blue arrows in donor B) were evenly distributed. Lymphatic endothelial cells formed larger capillaries (CD31+ low/LYVE‐1+, predominantly green in donor B and red in donor C, delimited with dashed lines) compared to blood capillaries (CD31+ high, red, indicated by red arrows). Macrophages or dendritic cells (CD206+, green in donor C) were observed near the capillaries, exhibiting a star‐like morphology with extensions. Scale bar: 100 μm. Images are representative of donors A–C. Figure S2: Growth of keratinocytes and fibroblasts from donors A–C cultured on plastic through 6 days. Growth curves show exponential growth in A. keratinocytes and C. fibroblasts. After 96 h, the cells have entered stationary phase. Doubling time calculations of B. keratinocytes and D. fibroblasts revealed slight significant differences in growth rates between donors. Statistical significance was assessed using one‐way ANOVA: *p < 0.05; **p < 0.01; ***p < 0.001. N = 3 donors, with three technical replicate [file WRR-34-0-s001.docx]

**An autologous, vascularized and immunocompetent Tissue Engineered Skin to highlight inter-individual variability to better understand the human wound healing**

Emilie Attiogbe, PhD^1,2,†^, Elodie Mareux, PhD ^1,2,†^, Sébastien Larochelle, Msc^2^, Adèle Mauroux^3^, Sandrine Gofflo^3^, Carine Mainzer^3^, Sylvie Bordes^3^, Brigitte Closs^3^, Caroline Gilbert, PhD^4,5^, Véronique J Moulin, PhD ^1,2,*^

^1^ Department of surgery, Faculty of Medicine, Université Laval, Québec, QC, Canada

^2^ Axe Médecine Régénératrice, Centre de Recherche du CHU de Québec-Université Laval, Québec, QC, Canada^3^ R&D department, SILAB, Brive, France

^3^ R&D department, SILAB, Brive, France

^4^ Axe Maladies Infectieuses et Immunitaires, Centre de Recherche du CHU de Québec-Université Laval, Québec, QC, Canada.

^5^ Département de Microbiologie-Infectiologie et d'Immunologie, Faculté de Médecine, Université Laval, Québec, Quebec, Canada

^†^ These authors contributed equally to this work

*** Corresponding Author:**

Dr. Véronique J. Moulin

LOEX Aile-R, CHU de Québec – Université Laval

1401, 18e rue

Québec, Québec, Canada

G1J 1Z4

Phone (418) 649-0252 #61715

Fax (418) 990-8248

veronique.moulin@fmed.ulaval.ca

**Keywords:** skin, wound healing, tissue engineering, 3D model, immune cells, microvasculature, autologous, inter-donor variability

## Supplementary material & methods

# Flow cytometric analysis

Flow cytometric analysis of the dermis extracted cells was performed using two panels at each institution and previously described^1^. The analysis was carried out usinga FACS Melody cytometer (BD Biosciences, Mississauga, ON, Canada) at LOEX and the I2MC cytometry platform (Inserm/UPS UMR 1297 - Institut des Maladies Métaboliques et Cardiovasculaires, Toulouse, France) at Silab. Data were analyzed with FlowJo V10.8 (BD Biosciences). Antibodies used for each laboratory are described in supplementary table S1. The first panel allowed to discriminate lymphatic endothelial cells (CD45-/CD31+/HLADR-), blood endothelial cells (CD45-/CD31+/HLADR+), leucocytes (CD45+), monocytes/macrophages (CD45+/HLADR+/CD14+) and dendritic cells (CD45+/HLADR+/ CD14-/CD1a+). The second panel allowed to identify T lymphocytes (CD31-/CD45+/CD3+), mast cells (CD31-/CD45+/CD117+) and fibroblasts/fibrocytes (CD31-/CD45-/CD34+ and CD31-/CD45-/CD34-).

# Growth Rate Evaluation

Keratinocytes from donors A-C were thawed and seeded at 5.0 × 10^5^ cells in 75 cm^2^ tissue culture flasks (Corning, Kennebunk, ME, USA) along with lethally irradiated human fibroblasts (iHF) (seeded at 6.0 × 10^5^ cells per 75 cm2 flask and cultured at least 7 days before the addition of keratinocytes) in complete medium (cDH) composed of Dulbecco's Modified Eagle's medium (DMEM Gibco™, Waltham, MA, USA) with 3:1 Ham’s F12 (Gibco™), supplemented with supplemented with 5% fetal calf serum (Wisent Inc.), 5 μg/mL of insulin (SAFC Bioscience, Lenexa, KS, USA), 0.4 μg/mL of hydrocortisone (Teva, Toronto, ON, Canada), 10 ng/mL of epidermal growth factor (R&D Systems, Oakville, ON, Canada), 0.212 mg/mL of isoproterenol hydrochloride (Sigma-Aldrich, Oakville, ON, Canada), 100 IU/mL of penicillin (Fresenius Kabi, Homburg, Germany), and 25 μg/mL of gentamycin (Galenova, Saint-Hyacinthe, QC, Canada). Fibroblasts from donors A-C were seeded at 5.0 × 10^5^ cells in 75 cm^2^ tissue culture flasks (Corning, Kennebunk, ME, USA) in DMEM supplemented with 10% fetal bovine serum, 100 U/mL penicillin G and 25 mg/mL gentamicin (Schering Inc., Pointe Claire, Canada). Keratinocytes and fibroblasts were harvested when they reached 80–90% confluence and seeded at 10^5^ cells per well in a 6-well plate (9,6 cm^2^/well) in triplicate in minimum medium (mDH) composed of DMEM (Gibco™, Waltham, MA, USA) with 3:1 Ham’s F12 (Gibco™), supplemented with 0.1% bovine serum albumin (Proliant, Ankeny, IA, USA), 0.1% fetal calf serum (Wisent Inc., St-Bruno, QC, CAN), 100 U/mL penicillin G (Sigma-Aldrich), and 25 µg/mL gentamycin (Gemini bio product, Sacramento, CA, USA). Keratinocytes and fibroblasts were harvested each day for 6 days, starting 24 hours after seeding (referred as Hour 0) and counted using a Beckman Coulter counter. The doubling time (D) was calculated on the basis of the culture time (Δt, in hours) during the exponential growth phase, and the number of cells at the beginning (N1) and at the end (N2) of this phase. The formula used was:

$$D= \frac{\ln\left( 2 \right)x\Delta t}{ln(\frac{N2}{N1})}$$

# Cell migration evaluation

Keratinocytes (P3; 5.0 × 10^4^ cells) from donors A-C were plated in triplicate (n = 3) in a 6-well plate (9,6 cm^2^/well) with 8.0 × 10^3^ iHF/cm2 in cDH. Fibroblasts (P3; 5.0 × 10^4^ cells) from donors A-C were plated in triplicate in in a 6-well plate (9,6 cm^2^/well) in cDH. A cross-shaped scratch was created on confluent cells in the middle of the plate using a 200 µl pipette tip (Sarstedt, Nümbrecht, Germany). This configuration allowed consistent positioning of the imaging field, ensuring standardized analysis across samples. Culture medium was immediately removed and rinsed three times with PBS at 37°C to remove detached cells and culture was continued in mDH. Bright-field microscope images (magnification of 40) were recorded on a digital camera at 0 (T0), 6 (T6), 18 (T18) and 24 hours (T24) post-scratch. A fixed area centered using the cross of the scratch was measured using ImageJ software (Wayne Rasband, National Institute of Health, Rockville, MD, USA) to quantify wound closure over time, following previous published instructions^2^.

## Supplementary figures and tables

|  |  |  |  | Endothelial cells (%) | | | Immune cells (%) | Fibroblasts (%) |
| --- | --- | --- | --- | --- | --- | --- | --- | --- |
| Laboratory | Donor | Age | Anatomical site | Total | BEC | LEC |  |  |
| LOEX | Donor A | 21 | Arm | 12.8 | 11.3 | 1.5 | 32.9 | 54.3 |
|  | Donor B | 44 | Abdominal | 13.1 | 9.5 | 3.6 | 20.7 | 66.2 |
|  | Donor C | 41 | Abdominal | 11.0 | 8.8 | 2.3 | 19.0 | 70.1 |
| Silab | Donor D | 50 | Mammary | 5.5 | 2.8 | 2.7 | 29.4 | 65.1 |
|  | Donor E | 58 | Mammary | 8.0 | 4.0 | 3.9 | 31.2 | 60.8 |
|  |  |  | Mean | 10.1 | 7.3 | 2.8 | 26.3 | 63.3 |
|  |  |  | SD | 3.3 | 3.7 | 1.0 | 6.4 | 6.0 |

**Supplementary Table S1: Flow cytometry characterization of dermal cells according to donors.** Dermal cell proportion following extraction for each donor by flow cytometry characterization made at the LOEX research center (Quebec City, Canada) and at the Silab’s research and development laboratory (Saint-Viance, France). BEC, Blood endothelial cells, LEC, Lymphatic endothelial cells. Results are expressed in percentage of total extracted dermal cells. Age in years.

|  | **Antibody** | **Panel** | **Supplier** | **Reference** | **Dilution (volume /millions of cells in 100µL)** |
| --- | --- | --- | --- | --- | --- |
| LOEX | CD1a-**APC** | 1 | BD Biosciences | 559775 | 10µL |
|  | HLA-DR-**BB700** | 1 | BD Biosciences | 745782 | 0.5µL |
|  | CD31-**PE** | 1-2 | BD Biosciences | 555446 | 10µL |
|  | CD14-**PE-Cy7** | 1 | BD Biosciences | 557742 | 20µL |
|  | CD45-**BB515** | 1-2 | BD Biosciences | 564585 | 2.5µL |
|  | CD117-**APC** | 2 | BD Biosciences | 550412 | 0.1µL |
|  | CD3-**PE-CY7** | 2 | BD Biosciences | 563423 | 1µL |
|  | CD34-**BB700** | 2 | BD Biosciences | 742246 | 0.1µL |
| Silab | CD34-**FITC** | 2 | Biolegend | 343504 | 7µL |
|  | CD31-**PE-Vio770** | 1-2 | Miltenyi | 130-110-671 | 5µL |
|  | CD45-**PerCP** | 1-2 | Miltenyi | 130-113-682 | 5µL |
|  | CD1a-**PE** | 1 | Miltenyi | 130-111-870 | 5µL |
|  | CD3**-APC-Vio 770** | 2 | Miltenyi | 130-109-464 | 10µL |
|  | HLADR-**PE eFluor 610** | 1 | eBiocience | 61-9956-42 | 4µL |
|  | CD14-**BV711** | 1 | Biolegend | 367139 | 12µL |
|  | CD117- **APC** | 2 | Miltenyi | 130-116-610 | 5µL |

# Supplementary Table S2: Antibodies used for cell characterization using flow cytometry.

| **Primary antibodies** | **Species** | **Reference (Company)** | **Dilution** |
| --- | --- | --- | --- |
| CD3 | Mouse IgG1 | 555330 (BD) | 1/500 |
| CD206 | Rabbit | 18704-1 AP (Proteintech) | 1/100 |
| CD45 | Mouse IgG2a | ab 30470 (Abcam) | 1/100 |
| LYVE-1 | Rabbit | 70R-LR006 (Fitzgerald) | 1/100 |
| CD31 (3D whole mount) | Sheep | AF 806 (R&D) | 1/100 |

# Supplementary Table S3: Primary antibodies used for cell characterization using immunofluorescence method.

| **Secondary antibodies** | **Invitrogen reference** | **Fluorochrome** | **Dilution** |
| --- | --- | --- | --- |
| Goat anti-mouse H+L | A11005 | AF594 | 1/100 |
| Donkey anti-sheep | A11015 | AF488 | 1/100 |
| Goat anti-rabbit | A11034 | AF488 | 1/100 |
| Goat anti-rabbit | A31733 | AF647 | 1/100 |
| Goat anti-mouse Ig1 | A21240 | AF647 | 1/100 |
| Goat anti-mouse IgG2a | A21241 | AF647 | 1/100 |
| Goat anti-mouse IgG1 | A21133 | AF546 | 1/100 |

# Supplementary Table S4: Secondary antibodies from Invitrogen used for cell characterization using immunofluorescence method.

| **Donor** | **Epidermal thickness (µm)** |
| --- | --- |
| Donor A | 215.18 ± 4.66 |
| Donor B | 152.07 ± 7.99 |
| Donor C | 161.74 ± 12.13 |
| Donor D | 172.83 ± 7.10 |
| Donor E | 200.28 ± 10.75 |

Supplementary Table S5: Epidermal thickness of aviTES 7-day post-wounding measured using Masson trichrome (donors A to C) or hematoxylin eosin saffron (donors D-E) staining.

**Supplementary Figure S1: Whole-mount immunofluorescence analysis of the unwounded aviTES**. Immune cells (CD45+, blue; indicated by blue arrows in donor B) were evenly distributed. Lymphatic endothelial cells formed larger capillaries (CD31+ low/LYVE-1+, predominantly green in donor B and red in donor C, delimited with dashed lines) compared to blood capillaries (CD31+ high, red, indicated by red arrows). Macrophages or dendritic cells (CD206+, green in donor C) were observed near the capillaries, exhibiting a star-like morphology with extensions. Scale bar: 100 µm. Images are representative of donors A–C.

Supplementary Figure S2: Growth of keratinocytes and fibroblasts from donors A-C cultured on plastic through 6 days. Growth curves show exponential growth in **A.** keratinocytes and **C.** fibroblasts. After 96 hours, the cells have entered stationary phase. Doubling time calculations of **B**. keratinocytes and **D.** fibroblasts revealed slight significant differences in growth rates between donors. Statistical significance was assessed using one-way ANOVA: **p < 0.05; **p < 0.01; ***p < 0.001*. N = 3 donors, with 3 technical replicate/donor and two independent experiments.


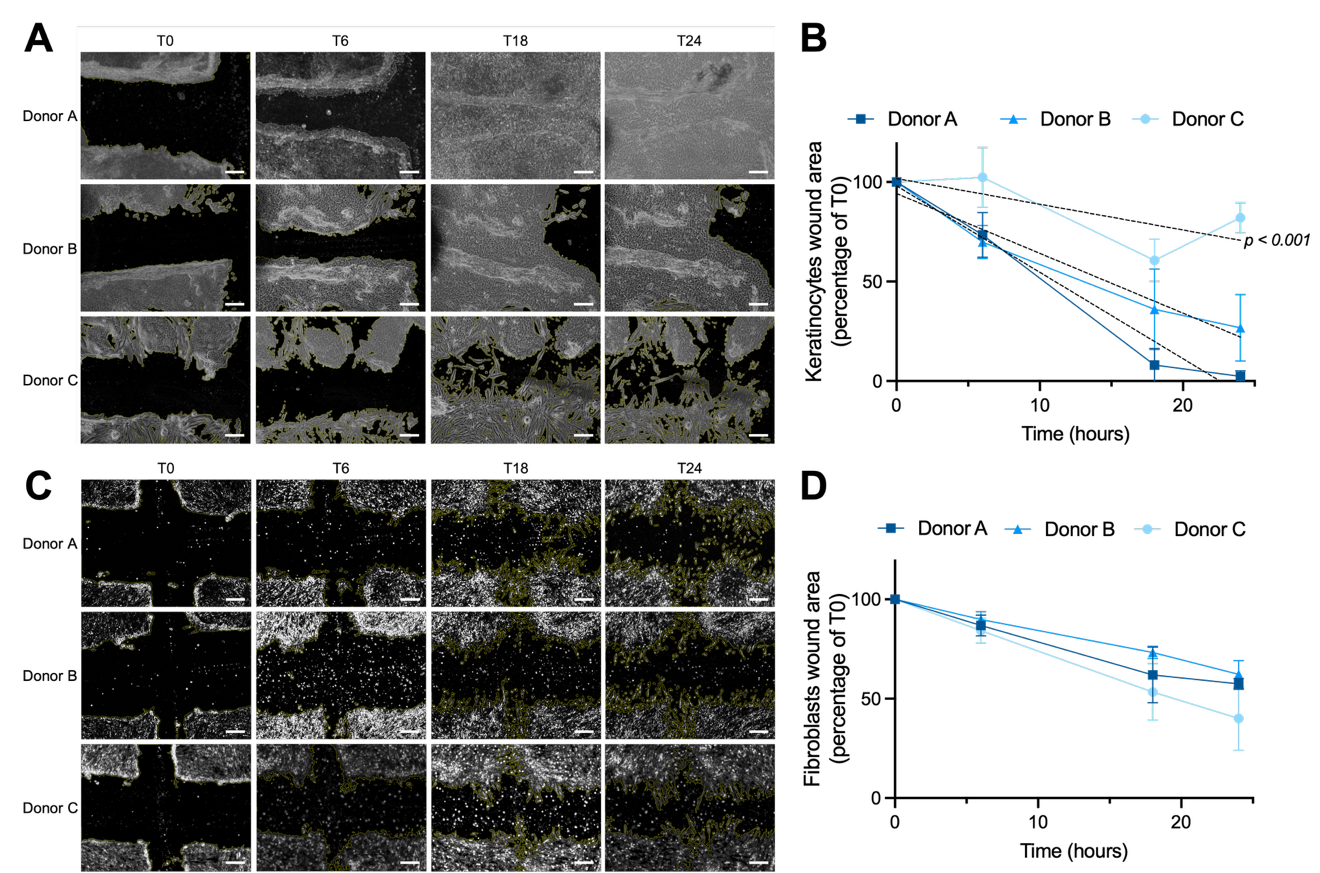


Supplementary Figure S3: Migration of keratinocytes and fibroblasts from donors A-C cultured in DH minimum. **A.** Keratinocytes and **C.** fibroblasts were grown until confluence then scraped with a 200 µl pipette tip. Cells were washed with PBS and replaced by DH minimum. Pictures were taken right after the scratch (T0) and at 6 (T6), 18 (T18) and 24 hours (T24). Scale bar: 200 µm. The migration rate of **B.** Keratinocytes and **D.** fibroblasts is represented as the percentage of the wound area. N = 3 donors with 3 technical replicate/donor and two independent experiments. The black dotted lines were included to represent the calculated slope of wound area reduction. The slope for keratinocytes from donor C was statistically different from the other donors.

Supplementary Figure S4: **Influence of Platelet Lysate (PL) treatment on blood and lymphatic networks in aviTES models**. Blood endothelial cells (CD31+^high^, red) and lymphatic endothelial cells (CD31+^low^/LYVE-1+, predominantly green) networks were visualized in the unwounded areas of the aviTES reconstructed with cells from 3 donors and cultured for 7 days either without PL (**A, B** and **C**) or with PL (**D, E** and **F**). Images are representative of 2-3 technical replicates/donors. Scale: 100 µm.

## Supplementary references

1. E. Attiogbe, S. Larochelle, Y. Chaib, C. Mainzer, A. Mauroux, S. Bordes, B. Closs, C. Gilbert, V.J. Moulin, An in vitro autologous, vascularized, and immunocompetent Tissue Engineered Skin model obtained by the self-assembled approach, Acta Biomaterialia (2023) S1742706123003781. <https://doi.org/10.1016/j.actbio.2023.06.045>.

2. Pijuan J, Barceló C, Moreno DF, Maiques O, Sisó P, Marti RM, Macià A, Panosa A. In vitro Cell Migration, Invasion, and Adhesion Assays: From Cell Imaging to Data Analysis. Front Cell Dev Biol. 2019 Jun 14;7:107. doi: 10.3389/fcell.2019.00107. PMID: 31259172; PMCID: PMC6587234.
